# Effects of Morphology Constraint on Electrophysiological Properties of Cortical Neurons

**DOI:** 10.1038/srep23086

**Published:** 2016-04-07

**Authors:** Geng Zhu, Liping Du, Lei Jin, Andreas Offenhäusser

**Affiliations:** 1 Institute of Complex Systems, Bioelectronics (PGI-8/ICS-8), Forschungszentrum Jülich, JARA – FIT, Jülich D-52425, Germany; 2 Bio-X Institutes, Key Laboratory for the Genetics of Development and Neuropsychiatric Disorders (Ministry of Education), and Shanghai Key Laboratory of Psychotic Disorders, Brain Science and Technology Research Center, Shanghai Jiao Tong University, 800 Dongchuan Road, Shanghai 200240, China; 3 Department of Biomedical Engineering, Zhejiang University, Hangzhou 310027, China

## Abstract

There is growing interest in engineering nerve cells *in vitro* to control architecture and connectivity of cultured neuronal networks or to build neuronal networks with predictable computational function. Pattern technologies, such as micro-contact printing, have been developed to design ordered neuronal networks. However, electrophysiological characteristics of the single patterned neuron haven’t been reported. Here, micro-contact printing, using polyolefine polymer (POP) stamps with high resolution, was employed to grow cortical neurons in a designed structure. The results demonstrated that the morphology of patterned neurons was well constrained, and the number of dendrites was decreased to be about 2. Our electrophysiological results showed that alterations of dendritic morphology affected firing patterns of neurons and neural excitability. When stimulated by current, though both patterned and un-patterned neurons presented regular spiking, the dynamics and strength of the response were different. The un-patterned neurons exhibited a monotonically increasing firing frequency in response to injected current, while the patterned neurons first exhibited frequency increase and then a slow decrease. Our findings indicate that the decrease in dendritic complexity of cortical neurons will influence their electrophysiological characteristics and alter their information processing activity, which could be considered when designing neuronal circuitries.

How neurons connect to each other to realize the function of the brain is one of the fundamental problems in neuroscience^1^,^2^. Experimental and computational methods have been used to study this issue. Experimental data collected by optical imaging and electrophysiological technologies shows that neurons present a wide range of morphology in different developmental periods and brain regions, as well as a diverse range of intrinsic electrophysiological firing patterns^3^,^4^. Differences in the type and distribution of ion channels over the neuronal membrane have been supposed as the reason for different firing patterns^5^. However, there is growing evidence that dendritic morphology affects neuronal activity. Using compartmental models of neocortical neurons with a common distribution of ion channels, Mainen and Sejnowski suggested the importance of dendritic geometry in neuronal function^6^. Later, some other studies also emphasized the role of dendritic morphology in determining the neuronal firing pattern^7^,^8^,^9^,^10^,^11^. Moreover, if the neuronal morphology could be manipulated precisely, it would provide direct experimental evidence to confirm the effects of dendritic geometry on neuronal function.

Engineering technologies, such as materials science and microlithography, have advanced to pattern cells with predefined geometry in culture dishes or on chips^12^,^13^,^14^,^15^. To pattern neurons, photolithography, soft lithography, electron beam lithography and other methods have been used^13^,^15^,^16^,^17^. Micro-contact printing (μCP), as a kind of soft lithography, is simple and cost-effective compared to photolithography and electron beam lithography. It can create sub-micrometer-scale chemical patterns on the solid substrate to constrain and guide the growth of neurons^13^,^18^. Poly-(dimethylsiloxane) (PDMS) was used to mold microstamps in the majority of μCP studies. However, PDMS has some serious drawbacks, such as low mechanical strength and homogeneity, when molding high precision forms. Recently, another kind of material, polyolefin plastomer (POP), with higher mechanical strength has been used to fabricate microstamps. Stamp production does not need to mix two materials before the imprinting process, increasing homogeneity. As such, POP microstamps have superior performance producing nanometer scale features. Furthermore, the results of the surface plasmon microscopy (SPM) demonstrated that the protein layer transferred by the POP microstamp is homogeneous and thick enough to guide the growth of neurons^19^.

In order to build a neuronal network with predictable function, it is essential to study not only the influence of morphology on individual neurons, but also the electrophysiological properties of such individually patterned neurons. Previous studies of patterned neuronal networks cultured on multi-electrode arrays (MEAs) have shown their different network activity compared to random dissociated cultures^20^,^21^,^22^,^23^. However, there are more than one neuron on an electrode, thus, it is still unknown how a single neuron plays a role in the network. A computational model made of meta-neurons was used to explain the dynamics of patterned neuronal networks on MEAs, and the simulated results showed the complexity of dendrites influenced the dynamics of single neuron activity and the whole network^11^. Patch clamp technique has been applied to patterned neurons, and the results demonstrated that the morphology restrictions would not impede the neuronal formation of different connectivity patterns^24^,^25^,^26^. However, dynamics of single patterned neurons’ activity haven’t been studied.

Here, we patterned the neurons one by one to form a simple circuitry. To realize the morphology constraint of single neurons’ dendrites, pattern geometry was designed according to a previous study that has manipulated the polarity of single neurons^27^. POP stamps were employed to print a one-node-and-three-line structure on the substrate in order to guide cortical neuronal growth. The morphology of a single neuron was constrained, and was characterized by immunostaining. Dynamics in patterned and un-patterned neurons were studied by patch clamp. The effect of morphological constraint on patterned neurons was compared to unpatterned neurons with regard to their electrophysiological characteristics by recording spontaneous and evoked electrical activities. Besides some common properties, we found some distinct electrophysiological characteristics between patterned and un-patterned neurons, which demonstrated that the dendritic structure is influencing the firing properties.

## Results

### Protein patterning

**Figure 1:**
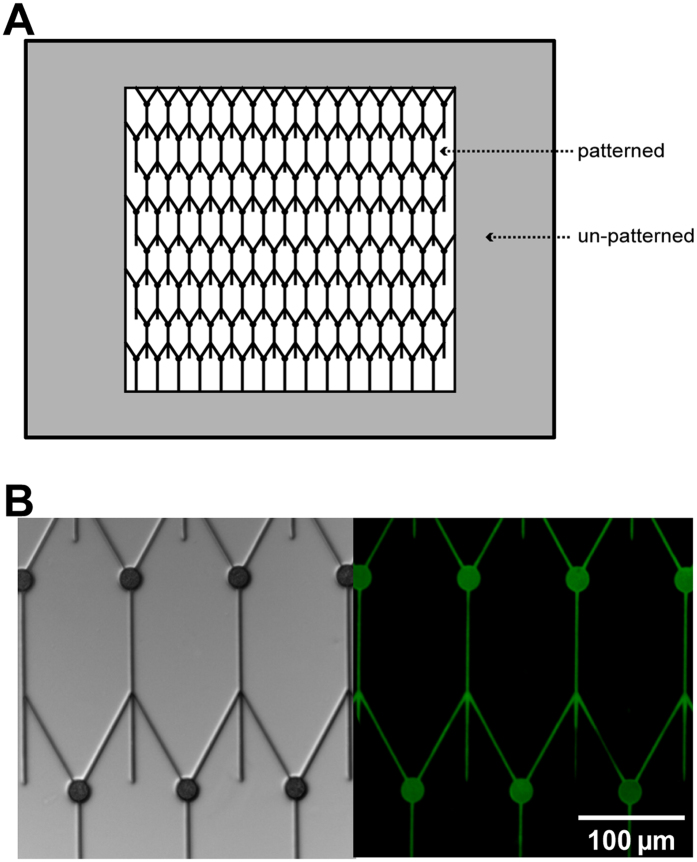
Design of POP stamps. (**A**) A draft of the design with un-patterned and patterned regions. (**B**) Left: DIC graph of a POP stamp shows what the morphology of neurons is supposed to be. The diameter of the nodes is 20 μm; the width of the lines is 2 μm, and the distance between two nodes is 100 μm laterally. Right: Corresponding PLL-FITC fluorescence micrograph shows that PLL was transferred to glass coverslips with the fine structures of the POP stamp. The scale bar represents 100 μm.

To study the influence of dendritic morphology on neuronal function, structures were designed as shown in Fig. 1, with two regions (patterned and un-patterned). The patterned and un-patterned neurons are from the same preparation and grow in the same micro-environment, which excludes the impact of the micro-environment on the electrophysiology of neurons.

In Fig. 1B, the DIC micrograph shows the designed structure of a POP stamp (left) was transferred with high precision, and the FITC-fluorescence image shows the PLL layer was homogenously transferred to the glass coverslip within the pattern (right). These results suggest that the POP stamp is very well suited to produce the high-quality printing for the constrained growth of neurons. Moreover, our previous study has demonstrated that the protein layer transferred by the POP stamp is thick enough for guiding the growth of neurons^19^.

### The morphology of patterned neurons

**Figure 2:**
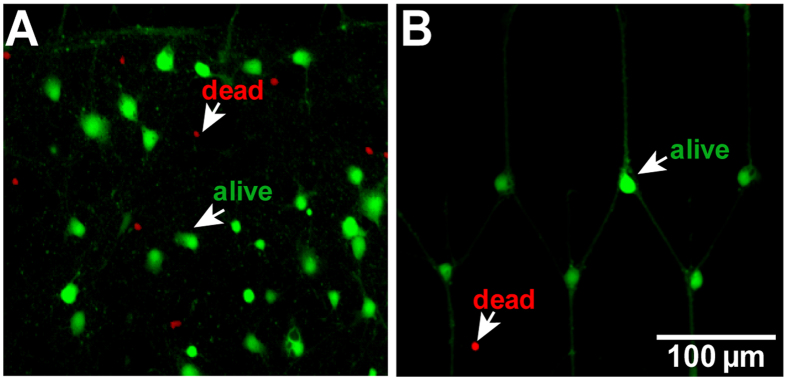
Cell viability of (**A**) un-patterned and (**B**) patterned neurons on DIV 8. Fluorescent images of neurons, green/red fluorescence for alive/dead cells. The scale bar represents 100 μm.

The growth of neurons was well controlled and constrained in the patterned region. To test the effect of morphological constraints on the survival rate of neurons, cell viability of patterned and un-patterned neurons at DIV 8 was determined using Calcein-AM and Ethidum homodimer-1/DNA for alive and dead cells, respectively. Typical fluorescent images are shown in Fig. 2. The survival rates of patterned and un-patterned neurons were 82 ± 2% (Mean ± SEM, n = 8) and 83 ± 2% (Mean ± SEM, n = 8), respectively. There was no significant difference between these two groups (two sample t-test two-tailed, p = 0.65), which indicated that the morphological constraint of neurite growth does not affect the survival of neurons at DIV8.

**Figure 3:**
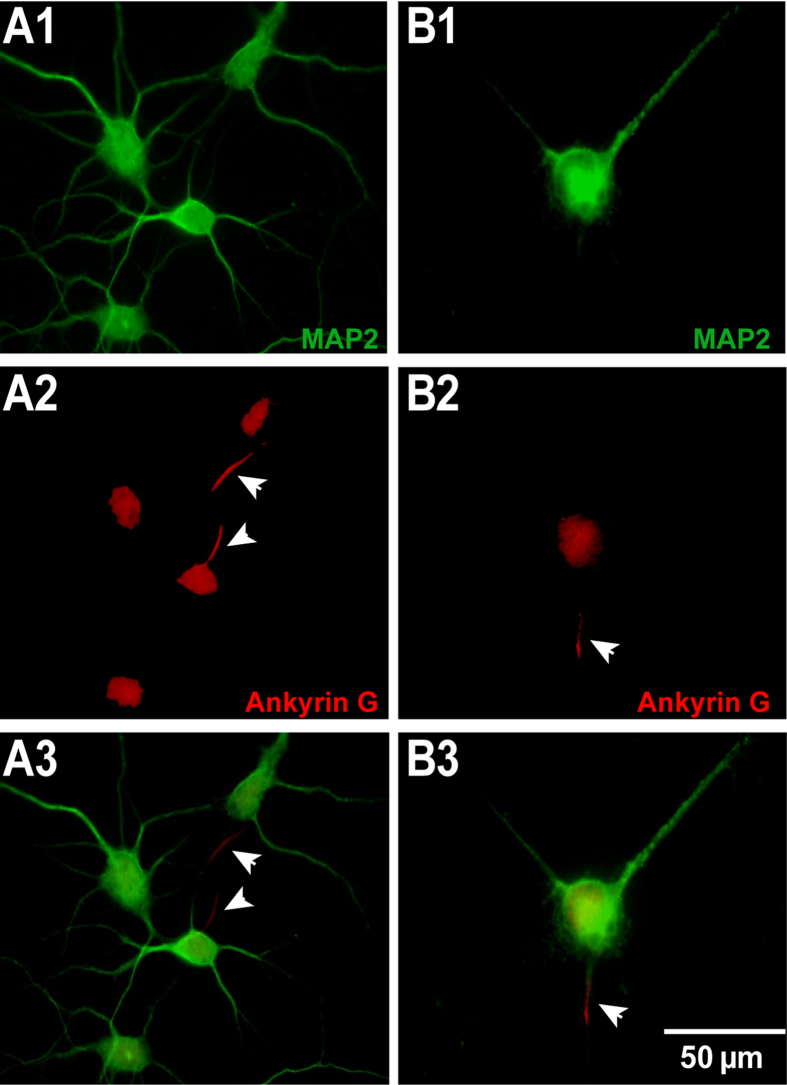
The morphology of (**A**) un-patterneds and (**B**) patterned neurons. Neurons were stained by MAP2 (green) and Ankyrin G (red) to indicate dendrites and axons, respectively. Un-patterned neurons have 3 ~ 8 dendrites (A1) and one axon for each neuron (A2). A patterned neuron has 2 dendrites (B1) and one axon (B2). (A3) and (B3) are the merged fluorescent images of MAP2 (green) and Ankyrin G (red). All the axons are indicated by arrows. The scale bar represents 50 μm.

Immunostaining was conducted to distinguish the axon and dendrite of neurons (Fig. 3). Therefore, the soma-dendrite area was stained by green (MAP2) and axonal initial segment was stained by red (Ankyrin G). For the un-patterned neurons, the neurites’ distribution is random in any direction. After arising from the soma, dendrites extend and branch multiple times. Here, the mean number of dendrites which sprout out of the soma at DIV 6 is 6 (n = 16 neurons) while the mean width of the first-order segment of such dendrites is 1.6 ± 0.1 μm (Mean ± SEM, n = 12 neurons), determined from immunofluorescence images. Each patterned neuron has neurites growing out in only three determined directions. In accordance with the work of Villard and colleagues^27^, our results show that neurons prefer (about 60%) to project the axon along the bottom line, as show in Fig. 3B. This was explained by the tension force theory that the axon would preferentially grow along the direction of highest tension^27^. In the other two directions, it was supposed to be one dendrite for each. We calculated the width of each first-order dendritic segment, and the mean width is 1.8 ± 0.1 μm (Mean ± SEM, n = 12 neurons). There was no significant difference in width of dendrite at the initial segment between un-patterned and patterned neurons (two sample t-test two-tailed, p = 0.07). Though there might be more than one dendrite patterned on one line, they were very close and their width is not significantly different from single dendrites, thus they were presumed to be one functional dendrite. Therefore, a patterned neuron has only three typical neurites, one is the axon and the other two are dendrites. This result indicates that patterned neurons, whose morphology was well controlled, extend a smaller number of dendrites.

### Electrophysiological results

**Figure 4:**
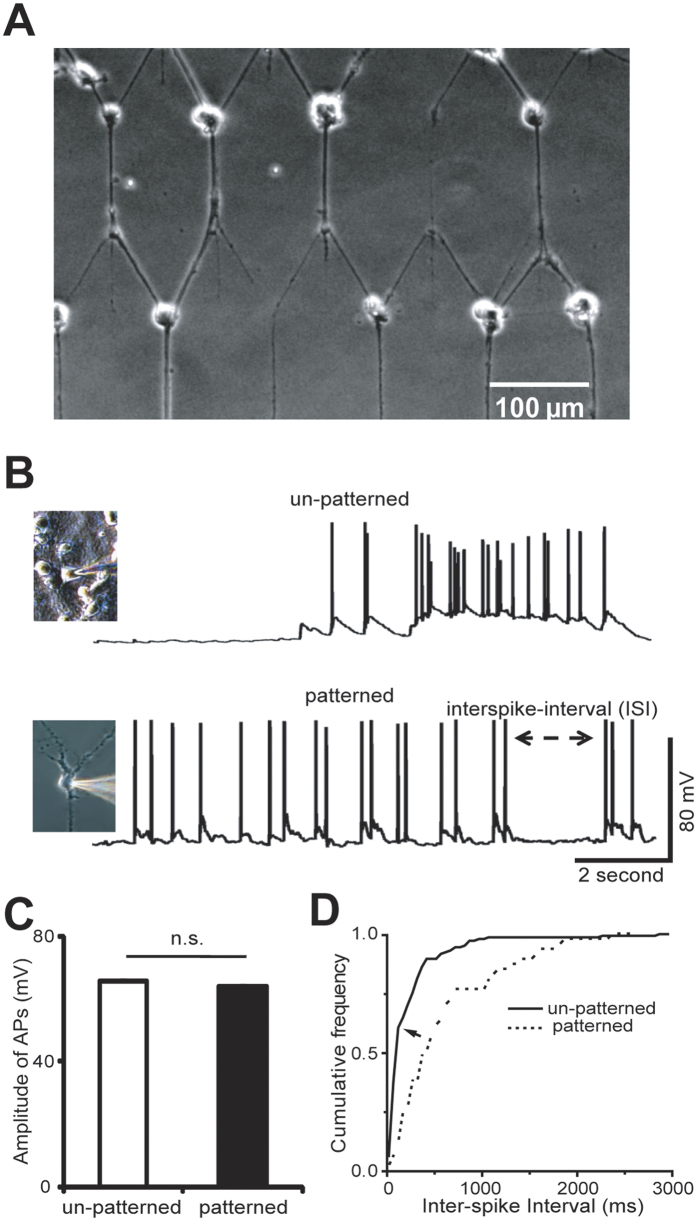
Spontaneous action potentials were recorded both from un-patterned and patterned neurons on DIV 14. (**A**) Phase contrast micrograph of patterned cortical neurons on a cover slip at DIV14. (**B**) Examples of 10 second raw data segment of an un-patterned and a patterned neuron. (**C**) There is no significant difference between amplitudes of action potentials of un-patterned (spikes from 7 neurons) and patterned (spikes from 7 neurons) neurons, using Student *t* test. (n.s., no significant difference) All data points represent mean ± SEM. Error bar indicates SEM.) (**D**) Cumulative frequency of ISI (Inter-spike intervals) of un-patterned (n = 7, black line) and patterned (n = 7, dashed line) neurons.

After 2 weeks cultured *in vitro*, alive neurons still maintained the pattern as shown in Fig. 4A. Cells were seldom alive off the pattern, due to the presence of FOTCS. Spontaneous action potentials were recorded by patch clamp at DIV 14. As shown in Fig. 4B, spontaneous spike trains were recorded from patterned and un-patterned neurons in whole-cell patch mode. Here 7 un-patterned neurons and 7 patterned neurons from 7 coverslips, which were cultured from 3 different preparations, were studied. The average resting membrane potential of patterned neurons was −60.1 ± 5.5 mV (Mean ± SEM, n = 7), and un-patterned cells was −56.4 ± 2.2 mV (Mean ± SEM, n = 7). The threshold level for spike detection was set at −40 mV. The results show that the spontaneous action potentials are similar in patterned and un-patterned neurons. The mean spike amplitudes of spontaneous action potentials are 66.0 ± 3.4 mV (Mean ± SEM, n = 7 neurons) and 63.9 ± 1.1 mV (Mean ± SEM, n = 7 neurons) for patterned and un-patterned neurons, respectively. There was no significant difference between each other as shown in Fig. 4C (two sample t-test two-tailed, p = 0.57).

However, the spontaneous firing patterns are different. The patterned neurons had regular-spiking firing patterns, while the un-patterned neurons had clustered-spiking (or bursting) firing patterns. In the cumulative frequency curve of inter-spike interval (ISI) as shown in Fig. 4D, the slope of short ISI (<150 ms) of un-patterned neurons is much higher than that of patterned neurons, which means there are a higher proportion of short ISIs in the firing pattern of un-patterned neurons. Regular spiking and bursting were two types of intrinsic neuronal firing patterns. The spontaneous regular spiking usually happened in the early stage of neuronal developments, while the spontaneous bursting happened after two weeks *in vitro* after neurons have established numerous synaptic connections between each other in a network^28^. Bursting is an alternation between an active phase of rapid spiking and a quiescent phase without spiking, and supposed to play diverse roles in neural computation^29^. There are lots of studies on the mechanism of bursting^30^,^31^,^32^,^33^, and various factors are supposed to correlate with bursting. It was reported that bursting patterns are correlated to neural development or the change of recurrent network connections^34^,^35^. Here, the neurons were on the same day *in vitro* in the same culture. In un-patterned circuits there were more recurrent connections found than in patterned circuits, which may be the reason why the un-patterned and patterned neurons fired in different patterns.

**Figure 5:**
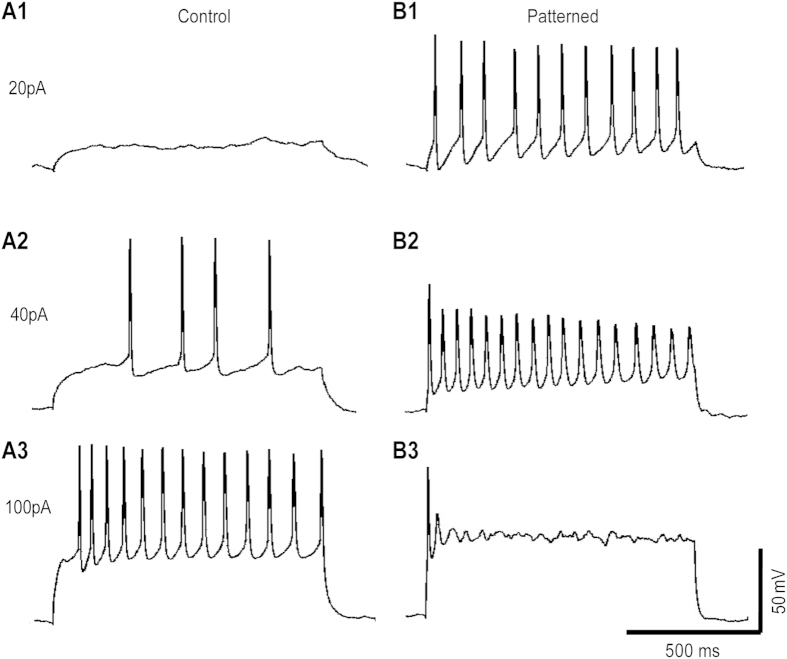
Action potentials were evoked using 1 second depolarizing current injections (20 pA, 40 pA, 100 pA). (**A**) A representative un-patterned neuron, a 20 pA current pulse did not evoke any action potential; a 40 pA current pulse evoked 4 action potentials; a 100 pA current pulse evoked 13 action potentials. (**B**) A representative patterned neuron, a 20 pA current pulse evoked 11 action potentials; a 40 pA current pulse evoked 17 action potentials; a 100 pA current pulse evoked 1 action potential and followed by a smaller spike.

To test the responses of these neurons to external stimulations, measurements in current clamp mode were conducted. Currents ranging from −20 to 180 pA were injected into neurons for 1 second. Compared to un-patterned neurons, patterned neurons can be stimulated to fire action potentials by lower current (20 ~ 40 pA), as shown in Fig. 5. When the injected current was 100 pA, the number of evoked action potentials in un-patterned neurons was maximum (8 ± 1.98 spikes, Mean ± SEM), with about 70% of neurons (5/7) firing regular spikes. When the amplitude of current was 40 pA, the number of evoked action potentials in patterned neurons was maximum (10.86 ± 2.36 spike, Mean ± SEM), again with about 70% of neurons (5/7) firing regular spikes.

**Figure 6:**
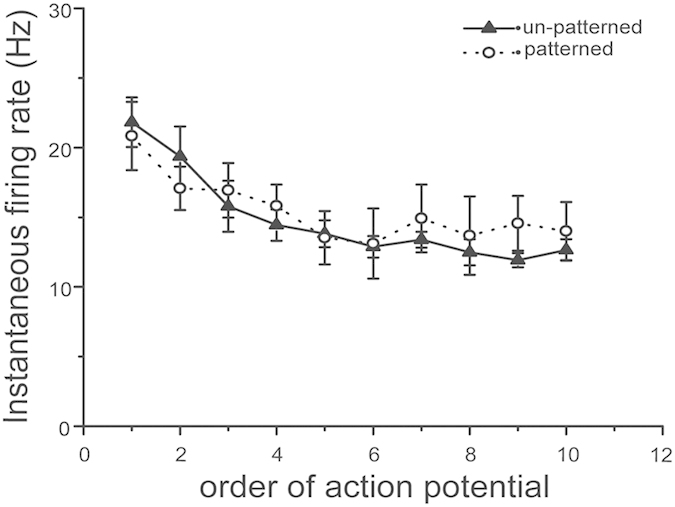
Firing patterns in un-patterned (▲) and patterned (○) neurons. Data was from 5 un-patterned neurons and 5 patterned neurons. Error bar indicates SEM.

The changes in firing rate of un-patterned neurons stimulated by 100 pA current and of patterned neurons stimulated by 40 pA current are shown in Fig. 6. The firing rate decreased until the 6th spike, and then kept this level, representing regular spiking, which is attributed to firing adaptation^28^. We calculated the ratio of firing rate of first spike and the 6th spike to describe the firing adaptation. The adaptation was 59.4 ± 1.9% and 71.3 ± 5.8% (Mean ± SEM, n = 5), for un-patterned and patterned neurons, respectively. Data was calculated from 5 un-patterned neurons and 5 patterned neurons from 5 cultures prepared on 3 different days were studied. There was no significant difference between groups (two sample t-test two-tailed, p = 0.09). Both un-patterned and patterned neurons were firing regular spikes with adaptation when responding to current stimulations. Previous studies reported that according to the patterns of action potentials generated in response to current injection, neurons of cortex can be divided into three types: regular-spiking (RS), fast-spiking (FS), and intrinsically bursting (IB) cells^4^. Here both un-patterned and patterned neurons show RS cell type behaviors.

**Figure 7:**
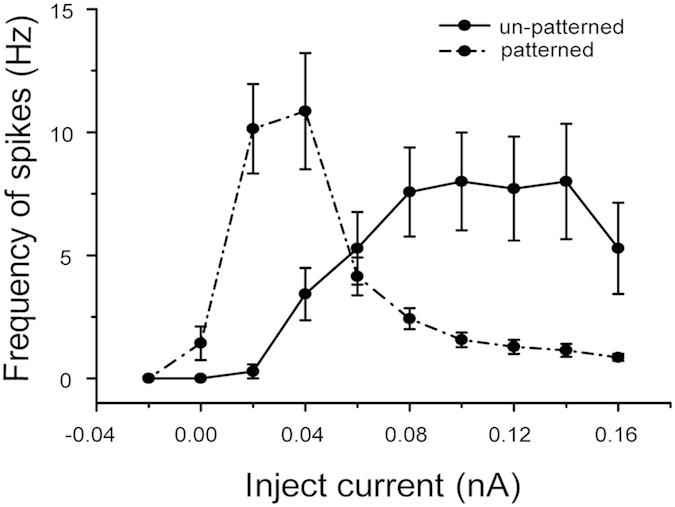
F/I curves of un-patterned and patterned neurons. The frequency of spikes was calculated from the number of spikes over the entire current pulse (1 second). Data was from 7 un-patterned neurons and 7 patterned neurons. Error bars indicate SEM.

Firing frequency was calculated from 7 un-patterned neurons and 7 patterned neurons from 7 coverslips prepared on 3 different days. According to frequency/injected current (F/I) curves (Fig. 7), patterned neurons showed different response dynamics from the un-patterned group. For patterned neurons, the firing rate first increased and then decreased when the injected current was increasing. However, the F/I curve of un-patterned neurons linearly increased at the beginning and then showed saturation at high firing frequencies, which is similar to previous reports^36^,^37^. The slope of patterned neurons’ F/I curve during its increasing phase was higher than that of un-patterned neurons, which means less current was required to elicit action potentials for patterned neurons. The increase in somatic Ri can reduce the amount of current required for spike induction. It was reported that the complexity of the dendritic tree could alter calcium dynamics^38^. Thereby, calcium-activated K^+^ channels may be involved in the decreasing phase of the patterned neurons’ F/I curve. Depolarization would normally induce enough rise in internal Ca^2+^, to activate enough calcium-activated K^+^ channels to repolarize the cell. In patterned neurons, altered Ca^2+^ may not activate a current large enough to repolarize the membrane, and then the total number of spikes was reduced.

In the patterned region, neurons had fewer dendrites and formed a less connected network, while in the un-patterned region neurons had more dendrites and formed a more complex connected network. Compared to un-patterned neurons, patterned neurons with fewer connections to other neurons showed higher excitability and encoded information by regular spiking.

## Discussion

In this study, POP stamps were used for micro-contact printing. PLL adhesive patterns were engineered to constrain the morphology of neurons. Our results demonstrated that the pattern of neurons can be maintained for more than 2 weeks, and the patterned neurons had fewer dendrites (2 dendrites) than un-patterned ones. It is not easy to keep such low density cultures alive *in vitro* for long term, compared to high density cultures, which can survive for more than one month. Cell culture technology for low cell density should be considered in the future application of patterned neurons.

Morphology constraints did not influence the short term viability of neurons (two weeks). The electrophysiological results revealed that both patterned and un-patterned neurons were RS cells, which may be pyramidal neurons or spiny stellates cells (about 80% cortex neurons). In accordance with a previous report^39^, the morphology constraint does not alter the amplitude of spontaneous action potentials. However, the intrinsic firing pattern and dynamic response to current injections are changed in patterned cortical neurons. The patterned neurons spontaneously showed regular spike firing patterns, and un-patterned neurons spontaneously showed bursting patterns. Moreover, the patterned neurons can be stimulated by lower current, and when stimulated by current lower than 0.05 nA, more spikes were induced in patterned neurons. These experimental results are in accordance with the simulation data reported by Massobrio and Martinoia^11^. When stimulated by higher current, patterned neurons presented less spikes. That may be because fewer calcium-activated K^+^ channels were activated, which normally prevents neurons from over-exciting. Therefor, our study provided direct experimental evidences to emphasize the importance of dendritic morphology in determining neuronal activity.

Patterned neuronal networks were cultured on MEAs in order to design a brain on a chip^23^. Specific network architectures to realize certain computational function can be designed by simulating based on neural models, and our pattern technology provides a way to draw such neuronal networks even with predictable connection direction. The agreement of our experimental and previous simulation data^11^ demonstrates that it is feasible to combine the computational and pattern technologies together to build neuronal networks with predictable computational function.

## Methods

### Micro-contact printing

Structures are designed to control the morphology of neurons as shown in Fig. 1A. There are two kinds of regions in each stamp, a patterned region in the middle and an un-patterned homogeneous region framing the pattern. Parameters of the pattern structure are critical for the successful neuronal patterning^40^. Here, the diameter of the node was set to be 20 μm to collect the soma of a neuron, as the average diameter of the soma of a cortical neuron is about 20 μm. Though 4 μm-line-width is suitable for neurite proliferation, it will result in more than one neurite grown on one line^39^. Therefore, the width of lines was set to be 2 μm to allow only one neurite to pass and control the number of neurites as precisely as possible. In the patterned region, each neuron is supposed to grow with a 20 μm-diameter soma, one 2 μm-wide axon (170 μm-long line), and two dendrites (two 90 μm-long lines with 60° angle). The distance between two neurons in a row is 100 μm, and between those in two columns is 160 μm.

POP stamps were produced by photolithography and casting. The structures were transferred into a dark field chrome mask by an electron beam writer. Master stamps were produced using a layer of positive photoresist (AZ5214E) on 0.6 mm thick silicon wafers (MEMC Electronic Materials, Germany), and followed by a baking step of 90°C for 5 min. The structures were transferred from the chrome mask to the resist in a mask aligner by exposure to the UV light for 4 s. Development of the resist was carried with MIF 326 (Karl Süss GmbH) for 50 s. After deep reactive ion etching with SF_6_ at 150 W for 10 min, a 1.90 μm deep structure was produced on the wafer. Finally, the mold was passivated by covalently linking a layer of (Tridecafluoro-1,1,2,2-tetrahydrooctyl) trichlorosilane (FOTCS) to its surface by a vapor deposition process to support the easy release of the plastomer. POP stamps were fabricated by sandwiching polyolefin polymer between the mold and a planar silicon wafer at 90°C for 5 min.

Poly-L-lysine (PLL) was transferred onto glass coverslips in designed structures by a μCP method. Firstly, glass coverslips were fully cleaned and activated in an oxygen plasma oven, and then silanized with FOTCS vapor at 45 mbar for 1.5 h. POP stamps were cleaned in 70% ethanol in an ultrasound bath and dried by a nitrogen stream, then immersed in the ink solution for 20 min. The ink solution was 10 μg/mL PLL (Invitrogen) diluted in Gey’s Balanced Salt Solution (GBSS) (Sigma). In order to evaluate the result of printing, fluorescein isothiocyanate (FITC) – conjugated PLL (PLL-FITC) (Sigma P3069) was used to visualize the printed pattern structure. After inking, POP stamps were dried in a nitrogen stream and pressed with an appropriate force onto the glass coverslip (Menzel, Germany) for 2 min.

### Cell culture

Cortical cells were dissociated from Wistar rats (embryonic day 18), and plated on PLL patterned glass coverslips with a density of 200 cells/mm^2^. Cells were cultured in Neurobasal medium (Invitrogen) with 1% B27, 0.25% L-Glutamate and 0.1% Gentamicin. The whole medium was replaced to remove unattached neurons 4 h after plating. Cells were maintained in an incubator at 37 °C in a humidified 5% CO_2_ atmosphere. Half of Neurobasal/B27 medium was changed every three days. All experiments were performed in accordance with §6 TierschG., §4 TSchG i.V. and §2 TierSchVerV, and all experimental protocols were approved by the Landesumweltamt für Natur, Umwelt und Verbraucherschutz Nordrhein-Westfalen, Recklinghausen, Germany.

### Live/dead staining and immunofluorescence staining

Cell viability was assessed after 8 days *in vitro* (DIV 8) by Calcein-AM (Invitrogen, Inc.) and Ethidum homodimer-1/DNA (Sigma). Numbers of alive and dead cells in un-patterned and patterned regions were counted separately. Survival rates were determined by dividing number of alive cells with total cell number (alive and dead cells).

Neurons were immunostained by MAP2 and Ankyrin G on DIV 6. Cells were fixed in 4% paraformaldehyde for 7 min at room temperature. After three times washing with 1×phosphate buffered saline (PBS), cells were blocked with blocking buffer (1% BSA and 2% goat serum in 1×PBS) for 1 hour at room temperature, and then permeabilized with 0.1% Triton X-100 for 15 min. After three times washing with 1×PBS, cells were incubated with primary antibody diluted in blocking buffer for 1 h at room temperature. Then, the respective secondary antibody was incubated for 1 h at room temperature. Finally, cells were washed two times with 1×PBS and one time with milliQ water, and mounted onto the glass slide by fluorescence mounting medium. Fluorescence images were captured by an Apotome system (Zeiss, Germany). Primary antibodies used here were anti-microtubule-associated protein 2 (anti-MAP2) (1:500, Milipore, AB5622) and anti-Ankyrin G (anti-AKG) (1:300, Life technologies, 33-8800). The secondary antibodies were Alexa FLuor 546 goat anti rabbit (1:500, Life Technologies A11035) and Alexa FLuor 633 goat anti mouse (1:500, Life Technologies A11030), respectively.

### Electrophysiology

Patch-clamp recordings were performed (EPC9, Heka Elektronik GmbH (Germany)) in the whole cell mode, at room temperature (air conditioned at 25°C). The culture medium was replaced with the pre-warmed (37°C) extracellular recording medium. Then neurons were washed twice, and incubated in 2 ml of the extracellular recording medium. The extracellular recording medium contained (mM): KCl, 3; NaCl, 120; MgCl_2_, 1; HEPES, 10; CaCl_2_, 2; pH 7.3 adjusted with 1 M NaOH (248 mosm/kg).

Borosilicate micropipettes (with filament and fire polished, Sutter, BF 150-86-10) were pulled by a micropipette puller (P-2000, Sutter Instrument Company, USA) immediately before each individual recording. Pulling parameters were set to obtain pipette resistances of about 8 MΩ. Before each recording, fresh pipettes were backfilled with intracellular pipette solution. The intracellular pipette solution contained (mM): KCl, 120; NaCl, 2; MgCl_2_, 4; HEPES, 5; EGTA, 0.2; Mg-ATP, 4; pH 7.34, adjusted with 1 M KOH (255 mosm/kg).

For current-clamp recordings, the membrane current was set to zero. Firstly, spontaneous firing activity was recorded for 10 seconds without any current injection. Then, a series of 9 current steps with 1 s pulse width in 500 ms intervals, ranging from −20, 0, 20, …, to 180 pA were applied to the cells and the resulting voltage signals were recorded.

The threshold for spike or action potential detection was set to −40 mV. The inter-spike interval (ISI) was defined as the time interval between two consecutive spikes. The firing rate of spikes evoked by current injection was calculated by the reciprocal of inter-spike interval.

## Additional Information

**How to cite this article**: Zhu, G. *et al*. Effects of Morphology Constraint on Electrophysiological Properties of Cortical Neurons. *Sci. Rep.* **6**, 23086; doi: 10.1038/srep23086 (2016).
